# Development of a Process for Separation of Mogroside V from *Siraitia grosvenorii* by Macroporous Resins

**DOI:** 10.3390/molecules16097288

**Published:** 2011-08-25

**Authors:** Min Zhang, Huihua Yang, Hongyang Zhang, Yuerong Wang, Ping Hu

**Affiliations:** 1 Modern Engineering Center for Traditional Chinese Medicine, School of Pharmacy, East China University of Science and Technology, Shanghai 200237, China; 2 College of Computer Science and Control, Guilin University of Electronic Technology, Guilin 541004, China; 3 School of Chemistry and Molecular Engineering, East China University of Science and Technology, Shanghai 200237, China

**Keywords:** mogrosides V, *Siraitia grosvenorii*, macroporous resins, separation, enrichment

## Abstract

A separation method was developed for the preparative separation and enrichment of the non-caloric sweetener mogroside V from *Siraitia grosvenorii*. The adsorption properties of six macroporous resins were evaluated. Results showed that HZ 806 resin offered the best adsorption and desorption capacities. Based on the adsorption experiments on HZ 806, the adsorption data were found to fit the Freundlich model well. The pseudo-second-order kinetic model showed the highest correlation with the experimental results. Separation was performed with deionized water and 40% aqueous ethanol solution as mobile phases. In a typical run, 100 g of herb was processed and 3.38 g of mogroside V with a purity of 10.7% was harvested. This separation method provided a 15.1-fold increase in the purification factor from 0.5% to 10.7%. The present study showed that HZ 806 resins were effective for the separation and enrichment of mogroside V from *S. grosvenorii*.

## 1. Introduction

The fruits of *Siraitia grosvenorii* (Swingle), Chinese name *Luohan Guo*, a herbaceous vine prevalent in China as well as in other Asian countries, have been used since antiquity to treat coughs, sore throats and constipation [1]. It is also commonly used as a non-caloric sweetener for food and beverages. Water extracts of *S. grosvenorii* were reported to be about 150 times sweeter than sucrose and remain stable even in boiling water for 5 h [2]. The over-consumption of food and beverages with high levels of sugar has been linked to diabetes [3], obesity [4], and dental carries [5]. Therefore, there is a huge demand for non-caloric natural sweeteners, while the safety of artificial sweeteners is always a concern of people. The unique properties of *S. grosvenorii* extracts, particularly their non-caloric feature and intense sweetness, could meet the needs of people in avoiding obesity, diabetes and dental carries.

**Figure 1:**
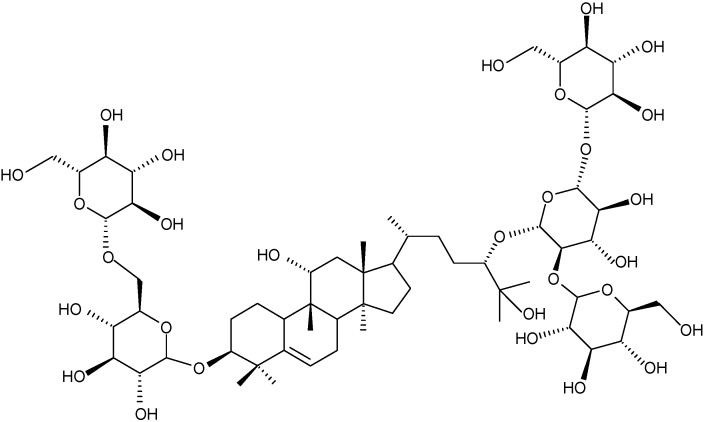
Chemical structure of mogroside V.

Cucurbitane-type triterpenoid glycosides are responsible for the taste associated with sweetness [6]. Among these components, mogroside V (Figure 1) is a sweetener found in relatively high amounts (0.50%) in *S. grosvenorii* [7]. The relative sweetness of mogroside V is about 250–450 times more intense than that of the same concentration of sucrose [8]. Mogroside V has also been reported to be an antioxidant agent that can scavenge reactive oxygen species and prevent DNA damage [9]. Therefore, there is increasing interest in developing methods with which to extract and isolate mogroside V from *S. grosvenorii* for either medical or food and beverage purposes.

Because herbs have complex components, the enrichment and separation of target compounds from herbal sources is considered to be a major issue by scientists. There is a growing need to develop efficient and selective separation processes. Several separation approaches, such as liquid-liquid extraction, membrane filtration, ion exchange, and adsorption, have become mainstream methods for herbal medicine separation, and macroporous resin (MR) adsorption is one of the most commonly used methods due to its low-cost, high-efficiency, easy-recycling, and simple scaling-up performance [10]. The principle of separation by MRs is based on molecule adsorption in differences of molecule polarity, weight, and size. Therefore, when selecting MRs for adsorption, their polarity, pore diameter, and surface area are the main concerns. Several studies on the applications of MRs [11,12,13] have shown the promising advantages of the MR separation of plant materials.

Several scientific articles have been published on the separation of mogroside V from *S. grosvenorii* using liquid chromatographic methods [14,15]. While previous studies employed various methods aimed at harvesting certain concentrations of the target compounds for bioactivity assays, no reports on MR selection, adsorption and desorption tests are currently available. Importantly, adsorption and desorption conditions are the most crucial issues when a separation by MR is set up. Moreover, such a separation process will be strongly welcomed if it can be scaled up from laboratory to pilot scale.

In the present study, we have attempted to set up a process for the separation of mogroside V from *S. grosvenorii* using MRs. The adsorption and desorption behaviors of the target compound were investigated on different resins, and the separation process was scaled up from the gram-scale to the hundred-gram-scale.

## 2. Results and Discussion

### 2.1. Selection of Macroporous Resins

**Figure 2:**
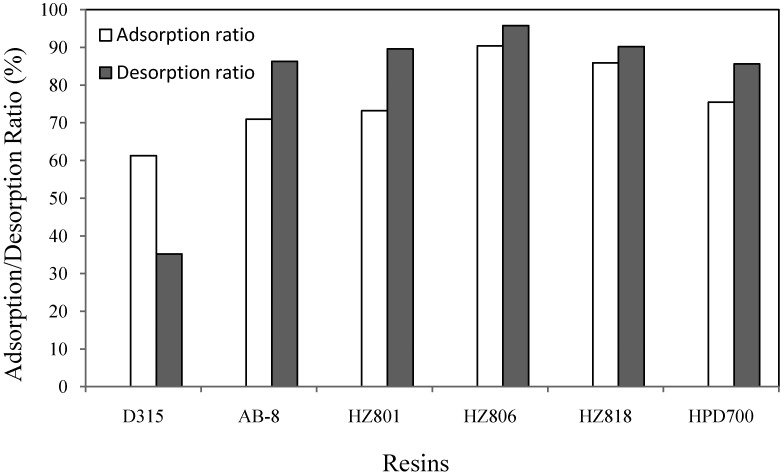
Adsorption/desorption ratios of mogroside V on different MRs.

Adsorption on MRs strongly depends on the match between target compound characteristics and resin properties. The characteristics of chemical targets include polarity and molecular weight, while the resin properties include polarity, surface area, and pore diameter. Non-polar resins can easily absorb non-polar substances in polar solvents, while polar resins can absorb polar targets from non-polar solvents. Several types of MRs, varying from non-polar resins to mid-polarity resins, were tested. The adsorption capacity and adsorption/desorption ratio results are shown in Figure 2.

According to Figure 2, HZ 806 had the highest adsorption ratio of 90.3%. This was due to the large surface area of these resins, as well as their matches to the target in polarity. The target, mogroside V, is classified as a mid-polar compound because of its chemical structure. In the present study, mogroside V was eluted with 33% of acetonitrile according to the HPLC analysis program described in Section 3.8, which further proved its mid-polar character. The desorption ratio was determined for the recovery of targets from extracted solution. Compared to the other resins, HZ 806 had the highest desorption ratio of 95.8%. Therefore, based on both adsorption and desorption properties, the mid-polarity HZ 806 resin was selected for further tests.

### 2.2. Adsorption Isotherms

Adsorption isotherms indicate the relationship between the amounts of target adsorbates distributed both in the adsorbents and in the solution at equilibrium. The Langmuir and Freundlich models are mostly used for describing adsorption isotherms. The Langmuir model is expressed as follows:

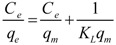
(1)
where *q_e_* (mg/g resin) is the adsorption capacity, *C_e_* (mg/mL) is the target concentration in the equilibrium solution, *K_L_* (mg/g) is the Langmuir constant, and *q_m_* is the empirical constant. *C_e_* and *C_e_*/*q_e_* were fitted to the Langmuir model to obtain constants.

The Freundlich model is given as follows:

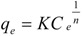
(2)

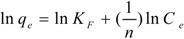
(3)
where *K_F_* is the Freundlich constant and *1/n* is an exponent that gives an indication of the favorability and capacity of the adsorption.

**Figure 3:**
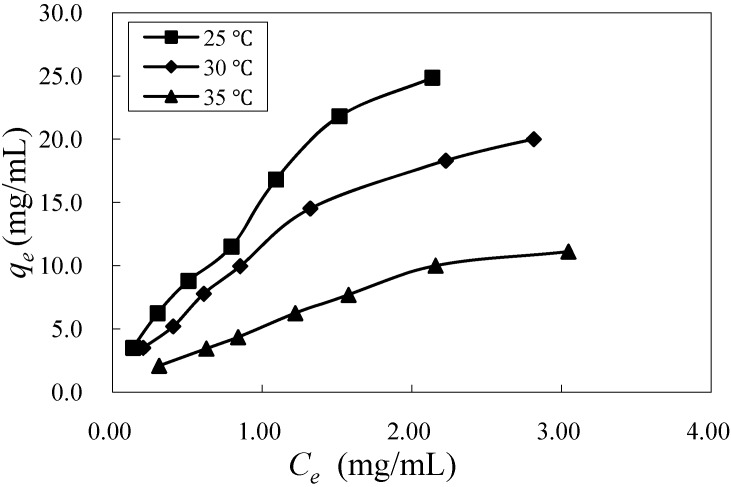
Adsorption isotherms of mogroside V on HZ 806 at different temperatures.

The adsorption isotherms of mogroside V at 25, 30, and 35 °C are shown in Figure 3. The isothermal data fitted to the two models were processed by curve fitting with Microsoft Excel Solver, and the fitted parameter values, which were graphically determined by linear regression, are presented in Table 3. As shown in Figure 3, the adsorption capacity of mogroside V at equilibrium decreased with increasing temperature.

The correlation coefficients of the Freundlich model were higher than those of the Langmuir model. Therefore, we conclude that the adsorption behavior was better explained by the Freundlich model. The exponent *1/n* shows the favorability and capacity of the adsorption, with an exponent between 0 < *1/n* < 1 showing beneficial adsorption. In Table 1, the values of *1/n* lying between 0 and 1 indicate that the adsorption of mogroside V on HZ 806 was beneficial.

**Table 1:** Isotherm parameters for mogroside V adsorption on HZ 806 at different temperatures.

Isotherm model	Temperature (°C)	Model parameters
Langmuir	25	*K_L_* = 0.9143; *q_m_* = 31.25; *R^2^* = 0.987
	30	*K_L_* = 0.6792; *q_m_* = 27.78; *R^2^* = 0.980
	35	*K_L_* = 0.3478; *q_m_* = 20.83; *R^2^* = 0.990
Freundlich	25	*K_F_* = 14.88; *1/n* = 0.734; *R^2^* = 0.993
	30	*K_F_* = 11.19; *1/n* = 0.757; *R^2^* = 0.996
	35	*K_F_* = 5.254; *1/n* = 0.742; *R^2^* = 0.991

### 2.3. Adsorption Kinetics

**Figure 4:**
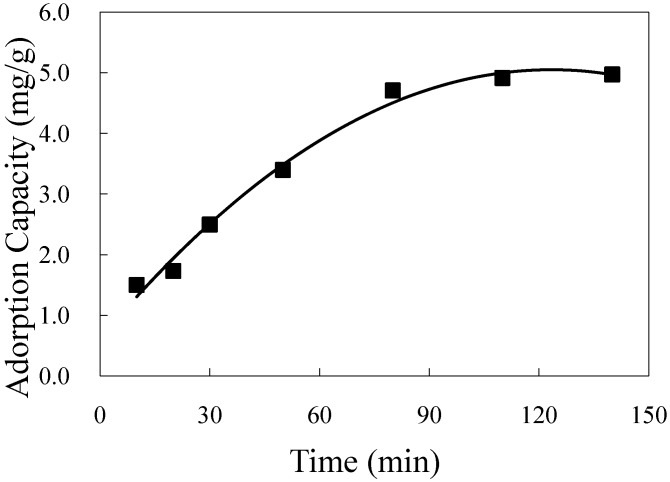
Adsorption kinetic of mogroside V on HZ 806 at 25 °C.

The adsorption kinetics of mogroside V on HZ 806 was obtained and is shown in Figure 4. The results showed that the adsorption capacity of mogroside V on the resins increased from 1.38 to 5.12 mg/g. The adsorption capacity was raised sharply during the 80 min, after which it increased slowly in the next 30 min. The adsorption capacity eventually reached equilibrium at 140 min.

To determine the rate of the adsorption process, two kinetics models, the pseudo-first-order and pseudo-second-order models, were used. The pseudo-first-order model is expressed as follows:

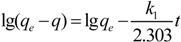
(4)
while the pseudo-second-order model is given as follows:

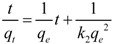
(5)
where *q_e_* (mg/g) and *q_t_* (mg/g) are the adsorption capacity at equilibrium and at time *t*, respectively. The parameters *k_1_* (min^-1^) and *k_2_* (g/mg min) are the rate constants of the pseudo-first-order and pseudo-first-order models, respectively. Kinetic equations and the parameters of the models were solved by Microsoft Excel Solver.

The kinetic parameters and correlation coefficients of the kinetic models are listed in Table 2. The *k_1_* and *q_e_* values of the pseudo-first-order kinetic model were determined from the slope and intercept of the plots of *lg (q_e_ − q)* against *t*, respectively. Although the correlation coefficient for the pseudo-first-order kinetic model was quite high (>0.9), the computed *q_e_* did not conform to the experimental value. Thus, the adsorption observed did not fit the pseudo-first-order model.

**Table 2:** Kinetic parameters for the adsorption of mogroside V on HZ 806 at 25 °C.

Kinetic model	Model parameters
Pseudo-first-order	*k_1_* = 0.0115; *q_e_* = 5.093; *R^2^* = 0.927
Pseudo-second-order	*k_2_*= 0.003; *q_e_* = 6.897; *R^2^* = 0.967

The plots of *t/q* against *t* for the pseudo-second-order kinetic had a linear relationship. The values of *k_2_* and *q_e_* were determined from the slope and the intercept. The value of computed *q_e_* was quite closer to its experimental value. Furthermore, the correlation coefficient (0.967) was higher than that of the pseudo-first-order kinetic. Therefore, the adsorption of mogroside V was favorably fitted using the pseudo-second-order kinetic model.

### 2.4. Breakthrough Point Determination on HZ 806 Resins

**Figure 5:**
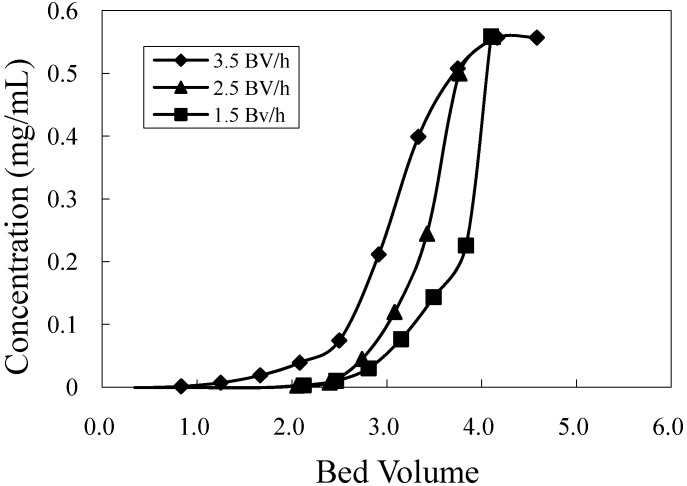
Dynamic breakthrough curve of mogroside V on HZ 806 resin.

The breakthrough point is used to determine of the maximum loading of the crude sample. Generally, an initial sample concentration of about 5% or 10% at the outlet is considered to be the breakthrough point [16,17]. In the present study, the breakthrough point was set as 10% of the crude sample concentration for a high throughput. The initial concentration of mogroside V was 0.56 mg/mL, and the flow rates tested were varied from 1.5, 2.5, and 3.5 BV/h. As shown in Figure 5, increasing the loading flow rate resulted in an early breakthrough point during adsorption. At a breakthrough point of 10%, the leakage of loading at a flow rate of 3.5 BV/h was observed at 2.1 BV, while the leakage point of 1.5 BV/h loading was found at 3.0 BV. This indicates that decreasing the loading flow rate by 71.4% will improve the loading capacity by 42.9%. Therefore, 1.5 BV/h was selected as the flow rate for loading.

### 2.5. Selection of Mobile Phase and Elution Flow Rate

Aqueous ethanol solution is usually used as the mobile phase during separation by adsorption resins. Different concentrations of aqueous ethanol solution, ranging from 0% to 40% (v/v), were used to test the elution capacity of the mobile phase. The selection of mobile phase was performed on a column packed with HZ 806 resins. Table 3 shows the elution abilities of different concentrations of aqueous ethanol. The desorption ratio of mogroside V increased sharply with increasing concentration of aqueous ethanol solution. The desorption ratio of the target compound reached to 98.0% when 40% aqueous ethanol solution was used as the mobile phase.

**Table 3:** Effect of the mobile phase on the mogroside V desorption ratio.

Aqueous ethanol solution (%)	Desorption ratio ^a^ (%)
0	0
10	0.02
20	67.7
30	86.0
40	98.0

^a^ The total amount of mogroside V applied to column is defined as 100%.

**Figure 6:**
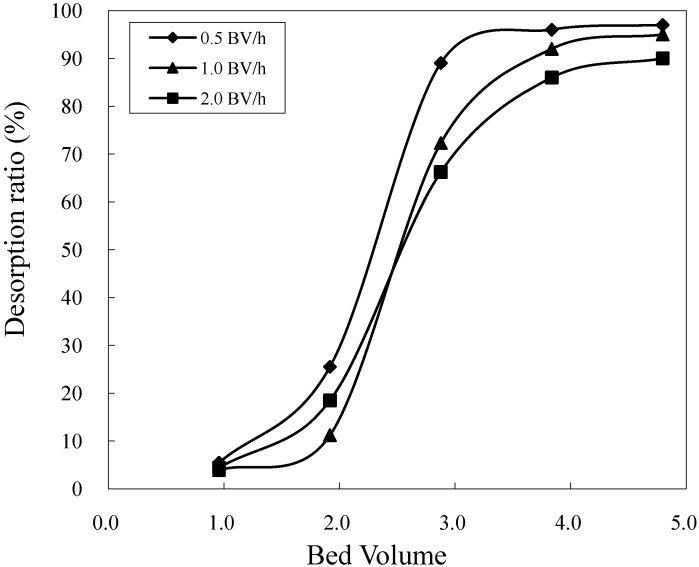
The effect of flow rate on desorption ratio.

The effects of flow rate on target desorption were investigated using an HZ 806 resin column. After loading the crude sample, elution was performed with 40% aqueous ethanol solution at flow rates that varied from 0.5 to 2.0 BV/h. Figure 6 illustrates the effect of flow rates on the desorption ratio. In all, 5.0 BV of the mobile phase was used to elute the target at different flow rates. The 0.5 BV/h flow rate showed the highest desorption ratio at 97.0%, while flow rates of 1.0 and 2.0 BV/h yielded desorption ratios of 95.2% and 90.0%, respectively. Flow rates of 0.5 and 1.0 BV/h were thus considered for succeeding elution.

Elution time is also an important factor for the optimization of elution conditions. At a lower flow rate, a greater proportion of the target compound is eluted from the chromatographic column; however, the separation process is more time-consuming. At a flow rate of 0.5 BV/h, the elution time was approximately 9.6 h, while, at a flow rate of 1.0 BV/h, the elution time decreased to 4.8 h. Therefore, in consideration of separation time, 1.0 BV/h was selected as the elution flow rate.

### 2.6. Separation of Mogroside V and Its Scale-Up

**Figure 7:**
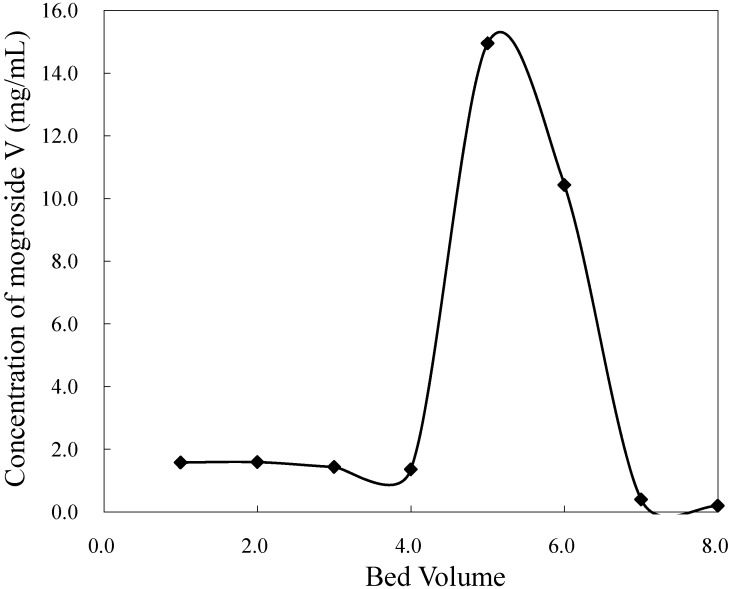
Elution profile of mogroside V on HZ 806 resin.

With the above optimized loading and elution conditions, separation of mogroside V was conducted on an HZ 806 resin column (200 mm × 10 mm I.D.). An amount of 15 g of *S. grosvenorii* was processed. Figure 7 shows a typical elution profile of mogroside V separation. Elution was performed with 2 BV of deionized water at a flow rate of 1.0 BV/h to remove contaminants. Afterwards, another 5 BVs of 40% ethanol was used to elute the target at the same flow rate.

**Figure 8:**
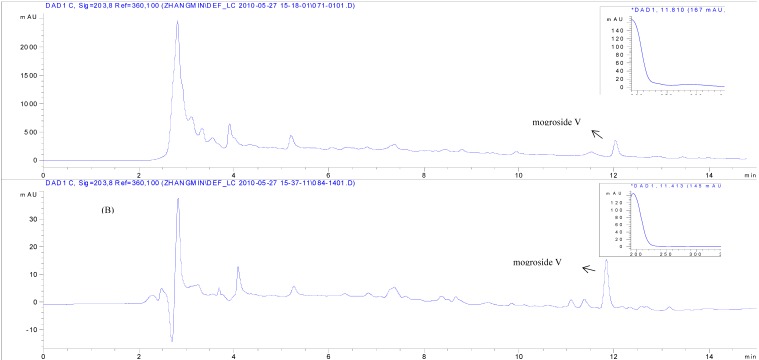
HPLC chromatograms of (A) *S. grosvenorii* and (B) purified mogroside V.

Separation was next transferred to the preparative scale and carried out on a 250 mm × 26 mm I.D. chromatography column. The extraction solution of 100 g of *S. grosvenorii* was loaded onto the HZ 806 resin column. The elution solvents, flow rate, and elution steps were the same as the above optimum conditions. Fractions were collected and analyzed by HPLC. The chromatograms of the extraction solution processed with HZ 806 resin are shown in Figure 8.

### 2.7. Process Evaluation

In the present study, a method for the separation of mogroside V from *S. grosvenorii* by HZ 806 resins was developed. An amount of 100 g of the herb was processed, yielding about 3.38 g of mogroside V with a purity of 10.7%. The method was evaluated by several process factors, including recovery (*R*), throughput (*Pt*), purification factor (*Pf*), and environmental factor (*Er*). Data used for the calculations were as follows: (i) initial content of mogroside V in the material was 0.5%; (ii) time for loading and adsorption was 1 h; (iii) time for elution was 7 h; (iv) time for regeneration of resins was 3 h; (v) purity of mogroside V in the product was 10.7%; and (vi) volume of solvent consumption was 1 L.

These factors were calculated by the Equations (9)–(12), and the results were: 


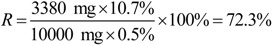



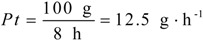



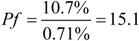



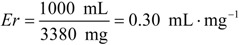


## 3. Experimental

### 3.1. Chemical and Reagents

Dried fruits of *S. grosvenorii* were supplied by a local farm in Guangxi Province, China. The content of mogroside V in herbs was determined as 0.5%. Mogroside V standard was obtained from the National Institute of China for the Control of Pharmaceutical and Biological Products (Beijing, China). Solvents used for separation were purchased from Sinopharm Chemical Reagent Company (Shanghai, China). Chromatographic grade methanol for HPLC analysis was supplied by the Tedia (Fairfield, NJ, USA).

### 3.2. Adsorbents

Different macroporous resins (HZ 801, HZ 806 and HZ 818) were provided by the Huazhen Science and Technology Company (Shanghai, China), HPD 700 was purchased from Bon-herb Technology Company (Hebei, China), and AB 8 was purchased from Chemical Plant of Nankai University (Tianjin, China). The physical and chemical properties of these different resins are listed in Table 4.

### 3.3. Preparation of Crude

The fruits of *S. grosvenorii* were dried in an oven at 105 °C and powdered. Five hundred g of this powder were extracted twice with boiling water (5 L) for 1 h each time. The extracted solution was combined, filtered and then concentrated to 500 mL by a rotary evaporator.

**Table 4:** Chemical and physical characteristic of resins.

Resins	Polarity	Surface area (m^2^/g)	Particle density (g/mL)	Particle diameter (mm)	Pore diameter (nm)	Moisture content (%)
HZ 801	non-polar	550	1.05–1.10	0.315–1.25	10.0	60–70
HZ 818	non-polar	900	1.0–1.10	0.315–1.25	9.0	60–70
HPD 700	non-polar	650-700	1.03–1.07	0.3–1.2	8.5–9	65–75
AB 8	weak-polar	480-520	1.05–1.09	0.3–1.25	13–14	60–70
HZ 806	mid-polar	600	1.02–1.12	0.315–1.25	12.0	68–78

### 3.4. Static Adsorption and Desorption

The selection of MRs began with static adsorption tests of mogroside V on HZ 801, HZ 806, HZ 818, HPD 700 and AB 8 resins. First, two grams of the different resins were placed in beakers. Second, 10 mL of the extracted solution was added into each beaker and mixed by shaking. These beakers were placed in a water bath for 12 h at a constant temperature. After that, 1 mL of the solution was transferred into a separate HPLC vial and analyzed by HPLC at 203 nm. After adsorption equilibrium was reached, the extracted solution was removed from the beakers by vacuum filtration. The remaining resins were desorbed with 20 mL of 95% ethanol for another 12 h. The desorption solution was then analyzed by HPLC. Different types of resins were scored by their adsorption capacities and desorption ratios. The evaluation parameters of capacity and ratio of adsorption were defined as:

Adsorption capacity:

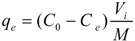
(6)


Adsorption ratio (%):

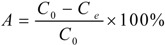
(7)
where *q_e_* is the adsorption capacity (mg/g resin), *A* is the adsorption ratio (%) at equilibrium, *C_0_* is the concentration of the target compound (mg/mL) in the initial extracted solution, *Ce* is the concentration of the target compound (mg/mL) in the equilibrated solution, *V_i_* is the volume of the extracted solution (mL), and *M* is the weight of the dry resins (g).

The evaluation parameters of desorption ratio were defined as:

Desorption ratio:

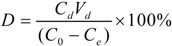
(8)
where *q_d_* is the desorption capacity at the adsorption equilibrium (mg/g resin), *C_d_* is the concentration of the target compound in the desorption solution (mg/g), and *V_d_* is the volume of the desorption solution (mL).

### 3.5. Adsorption Isotherm

Adsorption isotherms were recorded for selected HZ 806 resins. Seven batches of HZ 806 resin (2 g) were placed in beakers, and, to each beaker, 10 mL of different concentrations of the extracted solution (10%, 20%, 30%, 40%, 60%, 80%, and 100%) were added. The adsorption capacity of resins was compared in terms of the Langmuir and Freundlich models at 25, 30, and 35 °C.

### 3.6. Dynamic Adsorption and Desorption

Dynamic adsorption and desorption tests were carried out on an open tubular column (200 mm × 10 mm, I.D.) packed with HZ 806 resin with a bed volume (BV) of 15 mL. Extracted solution of *S. grosvenorii*, prepared using the method described in Section 3.3, was loaded onto the column with varying loading flow rates. Loading of the sample was ceased when the concentration of mogroside V at the outlet was the same as that in the extracted solution. 10% of mogroside V at the outlet was considered as the breakthrough point. The effect of loading flow rate on adsorption capacity was also investigated. Several variables were studied to determine their effects on the elution of the target compound. The target compound was eluted with different ratios of aqueous ethanol solution to test the solvents elution capacities. When the elution solvent was screened, elution conditions, particularly the elution flow rate and elution volume, were optimized.

### 3.7. Process Evaluation

In a typical separation process by MRs, the most commonly used parameters are target recovery and purity. Recovery is expressed through *R* as:

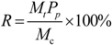
(9)
to show the ratio of the target compound harvested in a separation process, in which *M_t_* (g) is the mass of the target in the product, *M_c_* (g) is the mass of the target in the crude material, and Pp is the purity of the target in the product. A practical separation process is usually associated with time and capacity. Process throughput [15] is defined as:

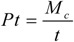
(10)
to evaluate mass processed per unit time, in which *Mc* (g) is the mass of the crude material and *t* (h) is the time spent per run. 

The purification factor is evaluated through *Pf*:

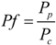
(11)
to compare the purity in the final product to the purity at the beginning source, in which *P_p_* is the purity of the target in the product and *P_c_* is the purity of the target in the crude herbal material.

The environmental risk factor [15] is described as:

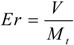
(12)
to calculate the volume of waste solvents produced during the separation process, in which *V* (L) is the total volume of the solvents used and *M_t_* (g) is the mass of the target compound harvested.

### 3.8. HPLC Analysis and Identification of the Fraction

The herbal material, crude extract solution, and isolated fractions were analyzed by HPLC at 203 nm. Analysis was performed on an Angilent 1100 series HPLC system equipped with a C_18_ analytical column (250 mm × 4.6 mm I.D., 5 μm, Agilent). Gradient elution was performed using water (solvent A) and acetonitrile (solvent B). The gradient program was as follows: 0–15 min, 15%–40% B; 15–16 min, 40%–15%; and 16–20 min held at 15% B.

## 4. Conclusions

A macroporous resins separation process for the separation and enrichment of mogroside V from *S. grosvenorii* was developed. HZ 806 resins were tested and found suitable for this separation. The experimental data fitted the Freundlich isotherm model well. The pseudo-second-order kinetic model provided the best correlation with the experimental results. One hundred g of *S. grosvenorii* herb was processed and 3.38 g of mogroside V with 10.7% purity were harvested. The method established has potential for the separation and enrichment of non-caloric sweeteners from herbal sources.
